# THE BLUES AND OLDER MINORITY MUSICIANS: MORE THAN JUST MUSIC XXX

**DOI:** 10.1093/geroni/igad104.1749

**Published:** 2023-12-21

**Authors:** John Migliaccio

## Abstract

That’s right! This GSA Annual Scientific Meeting celebrates 30 years of the ever popular “‘Bo Diddley’ Track” providing insights into the older performers who have pioneered and preserved this uniquely American musical genre and distinctive cultural treasure. Tampa was, and remains, a significant location in its history. When Hudson Woodbridge (Whittaker), a.k.a. “Tampa Red”, had learned all he could of the popular “race records” featuring legendary blues performers like Ma Rainey, Bessie Smith, and Ida Cox and moved to Chicago in 1925 in his early 20s, he brought with him a prolific expertise on the slide guitar and helped introduce multiple new forms of this music. He spread the popular bawdy and humorous “hokum” Blues style and created numerous classic tunes that are still covered by the most famous musicians of today. Tampa continues to nurture a thriving music scene, and this year’s GSA conference will take full advantage of the magic, music, and musicians of the Suncoast Blues. This year’s special celebration will start with the opening of the exhibit hall. Then continue with the popular traditional lecture, oral history, refreshments, photo op and mini-performance by the best and most resilient local older blues musicians. That evening, join the artist and band, and a multitude of fellow GSA members, for a lively music bash at a local club featuring dancing, local food and refreshments, swag, and one of the best parties at GSA. It’s a cornucopia of education and enjoyment like none other.

